# Density Dependence in Large Herbivores Inhabiting an Insular Nature Reserve

**DOI:** 10.1002/ece3.70689

**Published:** 2024-12-04

**Authors:** Cornelius J. Louw, Francesca Parrini, Jason P. Marshal

**Affiliations:** ^1^ Conservation Ecology Research Unit, Department of Zoology and Entomology University of Pretoria Hatfield South Africa; ^2^ School of Animal, Plant and Environmental Sciences, University of the Witwatersrand Johannesburg South Africa

**Keywords:** density dependence, insular reserve, large herbivores

## Abstract

While studies often focus on density‐dependent responses of ungulate populations to resource limitations at large spatial scales, the dynamics at smaller scales remain relatively unexplored. To address this gap, we investigated the temporal dynamics of ungulate abundance in a small dystrophic grassland ecosystem over 20 years, with minimal management interventions. We used annual counts and herd composition data to explore density‐dependent responses at the population level and attempt to link such responses to demographic mechanisms. Counts were corrected using a state‐space modeling approach. Populations of most species increased from low densities to approximate equilibrium densities. Our findings reveal evidence of density‐dependent responses in population growth that shaped the population abundance dynamics. Additionally, juvenile‐to‐adult ratios exhibited patterns of density‐dependent reductions in recruitment. This study suggests grassland ecosystems with moderate annual rainfall are regulated primarily by bottom‐up processes.

## Graphical abstract

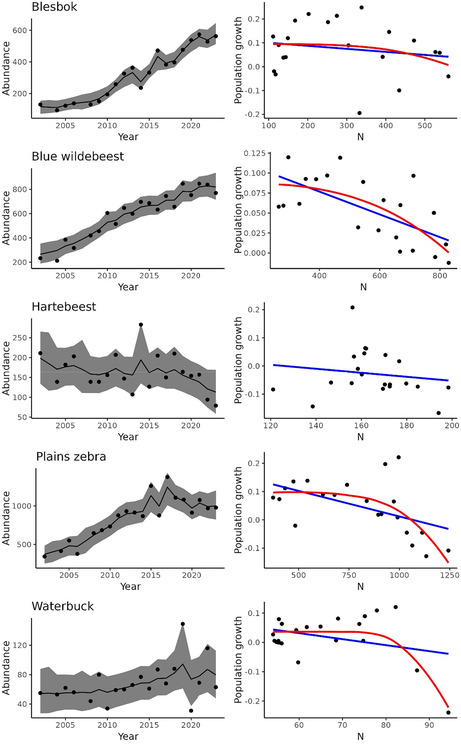

## 1 Introduction

Ungulate population dynamics are influenced by both density‐dependent and density‐independent processes (Royama 1992; Sinclair 2003). The significance of these processes is often contingent upon climatic factors (e.g., precipitation variation; Davis, Pech, and Catchpole 2002). Density dependence typically manifests through consumer‐resource coupling, leading to population regulation through increased mortality (DeAngelis and Waterhouse 1987; Ellis and Swift 1988; Sinclair and Pech 1996; Owen‐Smith 2002) and or predation (Hixon, Pacala, and Sandin 2002). However, its prominence varies across ecosystems. In northern latitudes, irruptive dynamics have been observed among a range of ungulate species (Gross, Gordon, and Owen‐Smith 2010), indicating the relative importance of density dependence, specifically bottom‐up regulation, in ungulate temporal dynamics (Sinclair and Pech 1996; Bonenfant et al. 2009). However, density dependence is less common in tropical and subtropical African savannas (Sinclair 2003; Owen‐Smith 2021). Frequent and prolonged droughts and highly seasonal environments result in ungulate populations commonly exhibiting disequilibrium dynamics (Illius and O'Connor 1999; Boone and Hobbs 2004; Derry and Boone 2010).

The relative importance of density dependence varies widely across space and time (McCullough 1999), influenced primarily by spatial and temporal heterogeneity of resources and climate variability (Wang et al. 2006). For instance, the Serengeti National Park wildebeest population has demonstrated resource‐mediated regulation following recovery from a rinderpest epidemic (Sinclair 1975; Sinclair, Dublin, and Borner 1985). Ungulate populations in Kruger National Park, South Africa, contrastingly, have exhibited significant fluctuations in abundance over time, highlighting the predominant role of climatic fluctuations (Owen‐Smith 2021). Consequently, the relative significance of density dependence and climatic variability in shaping the temporal dynamics of wildlife populations remains a relevant yet controversial topic (McNaughton and Georgiadis 1986). Consumer‐resource systems are best viewed as operating along a disequilibrium continuum (Wang et al. 2006), with varying frequencies at which density‐dependent responses have an overriding influence (McCullough 1999).

Density‐dependent growth responses for large herbivore populations become stronger with increasing population size (Sinclair 2003). In the absence of predators, the shape of these responses reflects the delay in negative demographic responses feeding back into population growth (Owen‐Smith 2010). The magnitude of over‐compensatory density dependence depends largely on the tolerance towards low‐quality forage during the dormant season (Owen‐Smith 2002), and perhaps especially so in dystrophic environments. While contingent on environmental conditions (McCullough 1999) the tendency for over‐compensation is commonly associated with generalist bulk feeders i.e., species of large body size (Fowler 1981). This perhaps particularly applies to species using a hindgut fermentation digestive strategy.

Zebra are large ungulates native to African Savanna and grassland ecosystems. Large body size, coupled with a bulk feeding strategy and hindgut fermentation, affords zebra greater tolerance to low‐quality forage so that negative demographic responses would be expected only close to equilibrium densities (Owen‐Smith 2002). Rising consumer densities reduce average forage quality (Hobbs 2024). Consequently selective feeders, in contrast to bulk feeders, likely experience resource limitations further from equilibrium densities under most conditions because of their relatively high energetic requirements (Owen‐Smith 2002).

This paper assesses the temporal dynamics and demographic responses of five large herbivore species, representing a range of feeding ecologies, in a 130‐km^2^ fenced conservation area. Unlike management policies common to small protected areas, which often involve frequent removals, those in Telperion Nature Reserve have involved minimal interference over the past two decades. Given the low variability in annual precipitation at the study site, we expected species population growth to be largely influenced by consumer density. We predicted the following: (1) all species would exhibit an over‐compensatory density‐dependent response typical of large herbivores (McCullough 1999; Sinclair 2003); (2) a theta logistic response in the zebra population as a consequence of its large body size (Fowler 1981); (3) consumer biomass dynamics levels off at equilibrium densities dictated by rainfall (Coe, Cumming, and Phillipson 1976); (4) zebra numerically dominate the ungulate assemblage given their greater tolerance towards low‐quality forage (Owen‐Smith 2002), while hartebeest (Murray and Brown 1993) and waterbuck (Becker et al. 2021) are numerically subordinate because of a lower preference for heavily utilized vegetation; (5) calf recruitment rather than the birth rate itself negatively correlates with consumer density (Gaillard et al. 2000).

## 2 Methods

### 2.1 Study Area

**FIGURE 1:**
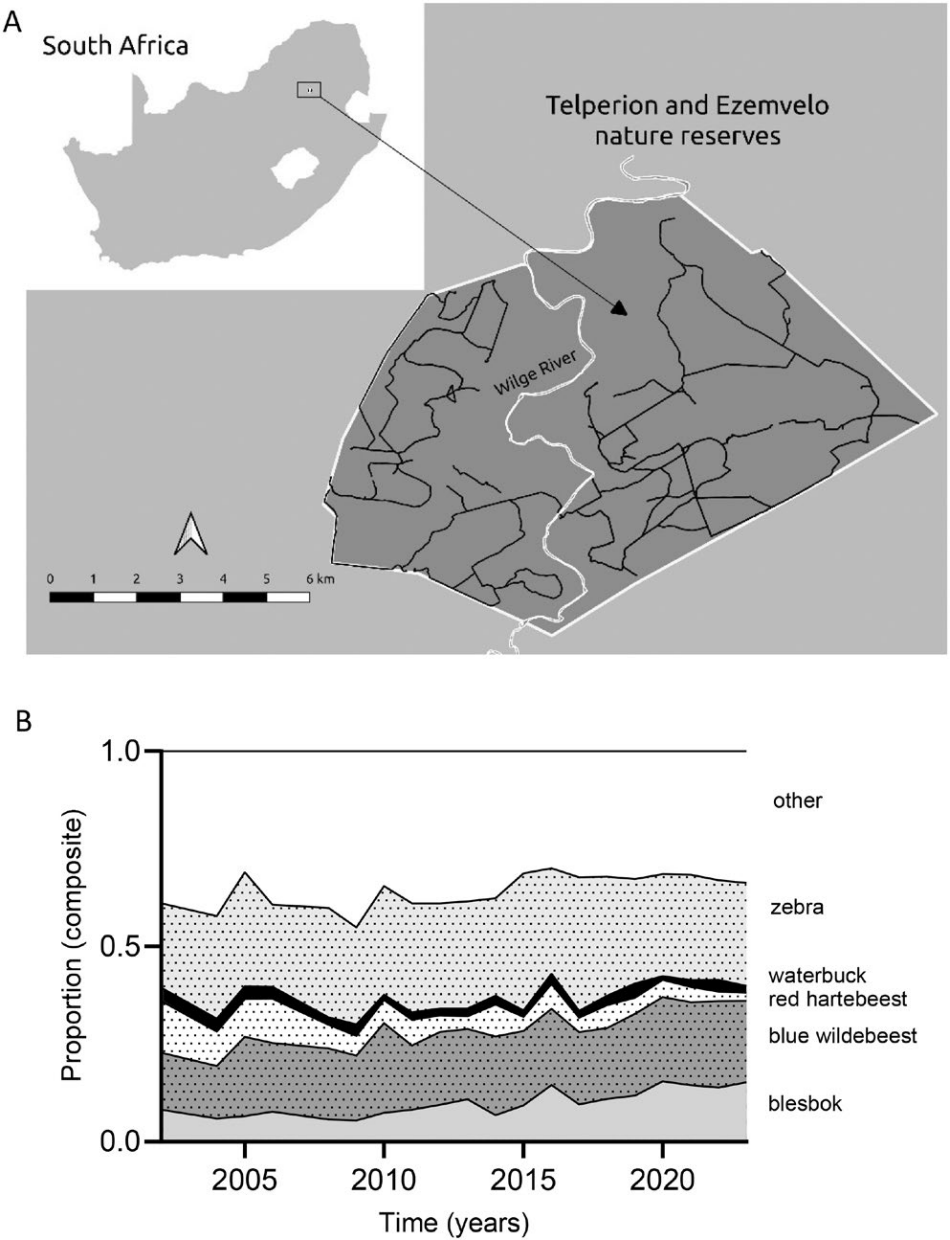
(A) Telperion and Ezemvelo nature reserves, straddling the border between Gauteng and Mpumalanga provinces, South Africa. To the west of the Wilge River (Ezemvelo), a lower altitude and deeper soil attracts higher densities of game, associated with more extensive grazing lawns relative to the area east of the Wilge River (Telperion section). The road network from which ground surveys were conducted is included. The more rugged terrain east of the river prohibited the construction of a more extensive road network. (B)Temporal changes in the proportional abundance of the study species at Telperion and Ezemvelo nature reserves during the study period from 2002 to 2023. “Other” represents a mixture of browser, grazer, and mixed feeder ungulate species.

Telperion and Ezemvelo Nature Reserves (25°38′ S, 29°03′ E), hereafter Telperion NR (Figure 1a), form a combined 130 km^2^ protected area within the grassland biome of South Africa (Coetzee 2012). The Wilge River separates the two sections but does not limit wildlife movement between them (pers. obs.). In addition, drainage lines and artificial dams, provide year‐round surface water to wildlife. Telperion NR receives an average annual precipitation of 654 mm, ranging between 570 and 730 mm (Coefficient of Variation, CV < 0.33) (Mucina and Rutherford 2006). The wet season spans from November to March, while the dry season goes from April to October. The Ezemvelo section, west of the Wilge River, is dominated by open grassland, whereas in the Telperion section, east of the river wooded grassland gradually replaces open grassland. Annual burning is carried out, with the Ezemvelo section experiencing more frequent burns than the Telperion section, which was previously managed separately. The area supports over 20 herbivore species, including blesbok ( *Damaliscus pygargus phillipsi* ), eland (*Tragelaphus oryx*), giraffe ( *Giraffa camelopardalis* ), impala ( *Aepyceros melampus* ), black ( *Connochaetes gnou* ) and blue wildebeest ( *Connochaetes taurinus* ), red hartebeest ( *Alcelaphus buselaphus* *caama*), waterbuck ( *Kobus ellipsiprymnus* ), and plains zebra ( *Equus quagga* ). The study species collectively comprise about 60%–70% of the overall ungulate community, with zebra being the dominant species (Figure 1b). Small carnivores, such as aardwolf ( *Proteles cristata* ), black‐backed jackal ( *Canis mesomelas* ), caracal ( *Caracal caracal* ) and leopards ( *Panthera pardus* ) also occur in the area. No reliable estimates of leopard density exist, but the low frequency with which spoor was encountered suggests densities are not particularly high. The study site is surrounded by commercial farmland where leopards get persecuted. Dispersal into the area is therefore limited by the Wilge River corridor that links Telperion with protected areas further afield.

The current owners bought the property in 1974, and in the mid‐1980s wildlife was (re)introduced. No hunting and or game removals have been conducted since then. This is unusual for protected areas of this size in South Africa. Aerial surveys commenced in 2002.

### 2.2 Data Collection

Annual total counts were conducted around early March each year since 2002, except in 2003 and 2007. Transects, 800 m wide, were flown along an east–west axis with a helicopter at an average altitude of 50 m. The study focused on five species: blesbok, blue wildebeest, red hartebeest, plains zebra, and waterbuck.

Herd composition data were collected once a month from 2011 to 2023, except for gaps from ~2019 to 2022, varying by species. Data were collected through ground surveys using a vehicle along fixed routes that remained unchanged over the study period (Figure 1). Demographic data analysis focused only on blesbok, red hartebeest, and blue wildebeest. The waterbuck population was too small for reliable data, and zebra are not birth‐pulse breeders, complicating the interpretation of young‐to‐adult ratios. We calculated young‐to‐adult ratios yearly for the December–January and February–March periods, where we pooled data for the December–January, and February–March periods respectively. To avoid the problem of differentiating between males and females, adult sex partitioning was not considered. This at least allowed a coarse assessment of recruitment with increasing consumer biomass. We assumed adult male survival rates to commence earlier than for females with deteriorating conditions (Gaillard et al. 2002). Such a prospect would increase young:adult ratios. Opposite to this, an observed decline in the young:adult ratio would thus lend credence to our results.

Monthly rainfall was obtained from eight weather stations spread across Telperion NR and averaged. Annual rainfall for a specific year was calculated from August 1st of that year (*R*_*t*_) until August 1st of the following year (*R*_*t*+1_).

### 2.3 Data Analysis

To estimate corrected abundances (*N*_*t*_) from observed counts (*y*_*t*_) for each species, we used a state‐space model (De Valpine and Hastings 2002; Buckland et al. 2004; Clark and Bjørnstad 2004) within a Bayesian hierarchical framework. The overall model consisted of sub‐models incorporating the ecological process representing abundances for each species, and the observation process generating uncorrected counts from those abundances (Kéry and Schaub 2012). Due to the likelihood of imperfect detection during aerial surveys and the absence of data to quantify this, the estimated abundances were interpreted as corrected indices of abundance (Kéry and Schaub 2012).

We used the state‐space model formulation adapted from Abadi et al. (2012), modified to remove the relationship with population density (Equation 1):

(1) $$N_{t+1}=N_{t}\times \exp\left[\log_{e}\left(\lambda_{t}\right)\right]$$

where *λ*_*t*_ = *N*_*t*+1_/*N*_*t*_, log_e_(*λ*_*t*_) was normally distributed with mean *r*_*t*_ and standard deviation *σ*_loge(*λt*)_, and *r*_*t*_ was defined as the maximum rate of change (*r*_*max*_). The prior distributions were defined to be noninformative: *σ*_loge(*λt*)_ was distributed as Uniform (0, 1) and *r*_*max*_ as Normal (0, 10,000).

For the observation model, counts *y*_*t*_ were distributed as Normal (*N*_*t*_, *σ*_*y*_), where *σ*_*y*_ was an estimate of the variability in the observation process. The prior distribution for *σ*_*y*_ also defined to be noninformative: Uniform (0.1, 1000).

We implemented the state‐space model in JAGS (Plummer 2003) running through R (R Core Team 2024) using package jagsUI (Kellner 2024). We used 3 chains of 1,000,000 iterations, discarding the first half of iterations as burn‐in, and we thinned to 1 value for every 100 iterations to reduce autocorrelation. We assessed convergence with the Brooks‐Rubin‐Gelman diagnostic $\hat{R}$ < 1.1 (Gelman and Shirley 2011). We also used visual assessment of trace plots and density plots to ensure that Markov chains converged and were not affected by parameters set for prior distributions.

Using the corrected estimates, we conducted a likelihood‐based analysis to assess the presence and form of density feedback on the population growth rate of each species by fitting geometric, Ricker, and theta‐logistic models. Following Fryxell, Sinclair, and Caughley (2014), the response variable was *r*_*t*_ = log_e_(*N*_*t*+1_/*N*_*t*_), and the explanatory variable for the Ricker and theta‐logistic models was *N*_*t*_.

- Geometric model:

$$r_{t}=r_{\max},$$

with *r*_*max*_ being a parameter to be estimated from the data

- Ricker model:

$$r_{t}=r_{\max}+b\times N_{t}$$

where *b* < 0 indicates density dependence.

- Theta‐logistic model: *r*_*t*_ = *r*_*max*_ × (1—*N*_*t*_ / *K*).

where values of 𝜃 > 1 represent a concave‐down density relationship commonly associated with overcompensatory population growth, and values of 𝜃 < 1 represent a concave‐up relationship indicating under‐compensation.

We fitted these models using the ‘*nls’* function and ranked them using AICc with the ‘AICcmodavg’ package (Mazerolle 2023) in R. The model with the lowest AICc value for each species was considered the best‐supported, and in cases where two models had similar AICc values, we interpreted both models.

To propagate the error from the corrected counts to the model parameter estimates, we fitted the best‐ranked model to each iteration of the corrected counts and rt. values from the saved Markov chains. This generated a distribution of values for each parameter based on the posterior distributions of the corrected counts. The point estimate for each parameter was the median for the resulting distribution of parameter values, and the 95% confidence limits were the 2.5% and 97.5% quantiles of those values.

To analyze temporal trends on overall grazer biomass, we used the mean body mass of wildlife species (Coe, Cumming, and Phillipson 1976). We also derived an equilibrium biomass for Telperion NR using the same model from Coe, Cumming, and Phillipson (1976), based on a time series of raw abundance estimates.

Herd composition data for blesbuck, wildebeest, and hartebeest were collected every month, where all individuals were partitioned into newborns and adults. To accommodate potential inexperience in field staff, we did not differentiate between sexes even though such partitioning existed in the data. Temporal trends in young:adult ratios were only qualitatively assessed due to gaps in the time series.

## 3 Results

### 3.1 Population Growth Models

**FIGURE 2:**
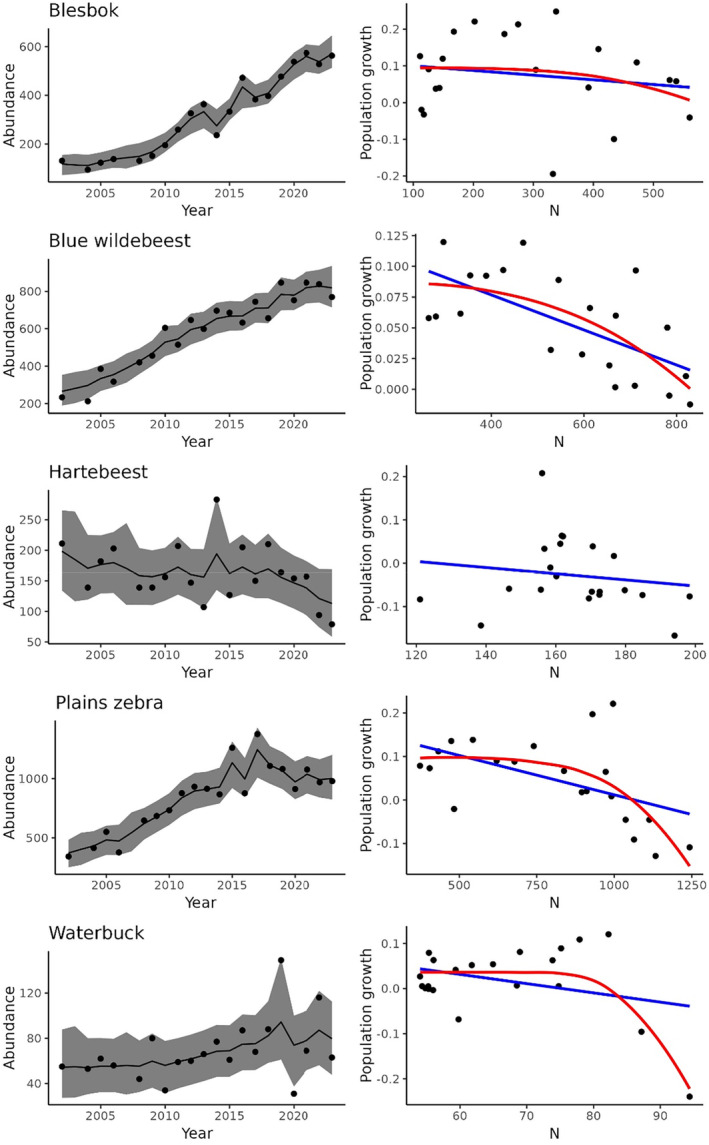
Time series plots of corrected counts of five ungulate species at Telperion Nature Reserve (2002–2023) along with a comparison between Ricker (blue line) and Theta‐logistic (red line) model fits. A theta‐logistic model is missing for hartebeest because of difficulties fitting the model for that species. The shaded areas represent the 95% credible intervals.

Blesbok, blue wildebeest, and zebra populations followed a distinct sigmoidal trajectory over time, while red hartebeest and waterbuck exhibited quasi‐equilibrium dynamics, with red hartebeest showing a decline towards the tail end of the study period (Figure 2). However, evidence of density dependence was found only in blue wildebeest, waterbuck, and, to a lesser extent, zebra populations.

For population growth, evidence of a concave‐down density relationship (𝜃 > 1) was observed in two species. The theta‐logistic model fitted the waterbuck data best (Table 1; Figure 2), while both geometric and theta‐logistic models were equally supportive of zebra data (Table 1; Figure 2). Linear density‐dependence (𝜃 = 1) was observed in blue wildebeest, with the Ricker model being the best fit (Table 1; Figure 2). Density dependence was not detected in blesbok and red hartebeest, with the geometric model best fitting their data (Table 1). Red hartebeest numbers changed little over time, while blesbok numbers increased four‐fold, hinting at a density‐dependent response towards the tail‐end of the time series (Figure 2). Parameter estimates for the best‐fitted models, and those closely matching these, are summarized in Table 2.

**TABLE 1:** Summary of model selection results for five large herbivore species at telperion nature reserve.

Model name	K	AICc	Delta AICc	AICc Wt	Cum. weight	LL
Blesbuck						
Geometric	2	**−35.51**	0.00	0.98	0.98	16.76
Ricker	3	−26.77	8.74	0.01	1.0	17.09
Theta‐logistic	4	−24.35	11.16	0.00	1.0	17.43
Blue wildebeest						
Geometric	2	−77.26	2.02	0.19	1.00	37.63
Ricker	3	**−79.27**	0.00	0.52	0.52	43.34
Theta‐logistic	4	−78.07	1.21	0.29	0.81	44.28
Red hartebeest						
Geometric	2	**−47.74**	0.00	0.99	0.99	22.87
Ricker	3	−38.81	8.93	0.01	1.00	23.11
Waterbuck						
Geometric	2	−49.67	7.04	0.03	1.00	23.83
Ricker	3	−42.41	14.30	0.00	1.00	24.91
Theta‐logistic	4	**−56.71**	0.00	0.97	0.97	33.60
Zebra						
Geometric		**−41.93**	0.00	0.45	0.45	19.96
Ricker		−38.89	3.04	0.10	1.00	23.15
Theta‐logistic		−41.91	0.02	0.45	0.90	26.20

*Note:* The bold values indicate the preferred models.

**TABLE 2:** Parameter estimates (median ± 95% credible intervals) of the best‐fitted models to time series of five large herbivore species at telperion nature reserve. Parameter estimates of models closely matching those of the best‐fitted model were also included.

Species	Model	Parameter	Median	95% CI
Blesbuck	Geometric	r_max_	0.07	0.06–0.10
Blue wildebeest	Ricker	r_max_	0.15	0.06–0.25
		b	< −0.01	−3.36 × 10^−4^—1.64 × 10^−5^
	Theta‐logistic	r_max_	0.09	0.05–0.27
		K	800.71	730.10–224.70
		theta	4.03	0.49–35.35
Red hartebeest	Geometric	r_max_	−0.03	−0.06—0.003
Waterbuck	Theta‐logistic	r_max_	0.06	0.01–1.32
Zebra	Geometric	r_max_	0.05	0.03–0.07
	Theta‐logistic	r_max_	0.11	0.07–0.22
		K	1054.70	968.13–1154.10
		theta	5.23	1.75–15.55

### 3.2 Consumer Biomass Trends

**FIGURE 3:**
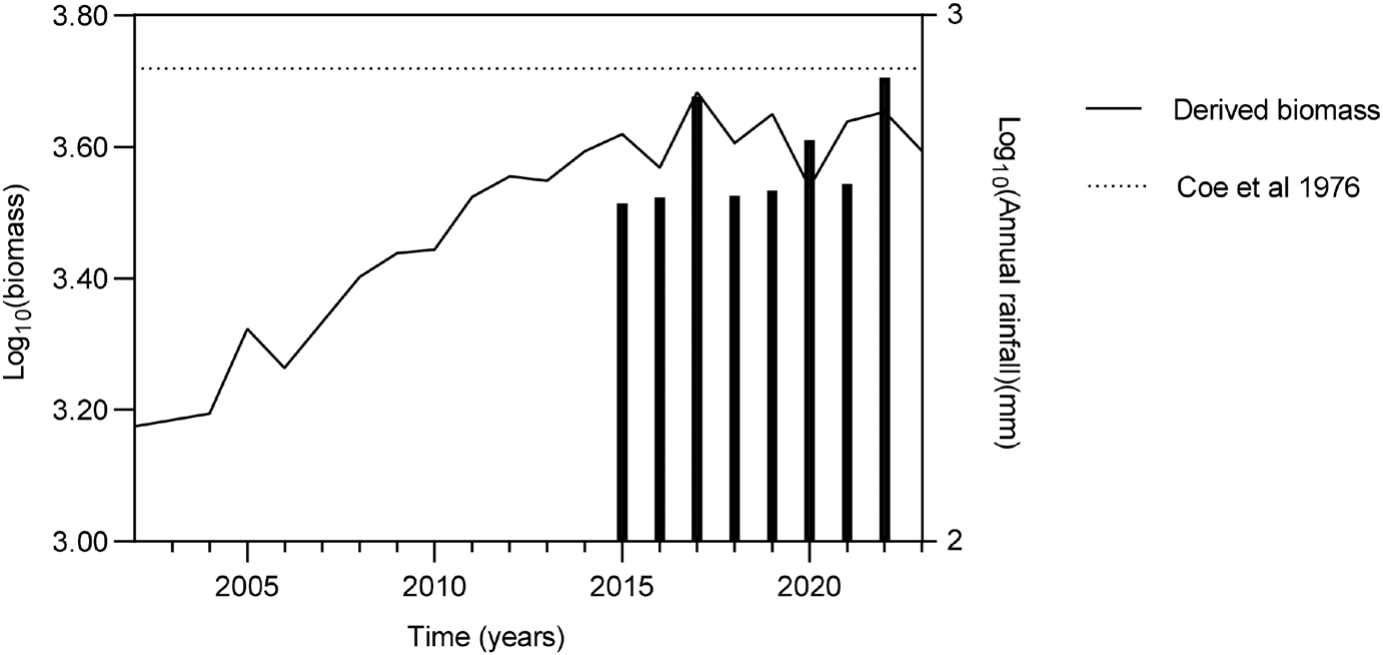
Temporal dynamics of overall consumer biomass of large herbivores, derived from raw count data and species body mass (Coe, Cumming, and Phillipson 1976) relative to equilibrium biomass predictions by the same author.

Zebra and wildebeest contributed the most to combined consumer biomass, followed by blesbok, hartebeest, and waterbuck (Figure 1). The mean annual rainfall for Telperion NR appears to support the model of Coe, Cumming, and Phillipson (1976) albeit the combined grazing herbivores biomass stabilized at densities somewhat below model predictions (Figure 3).

### 3.3 Demographic Responses

**FIGURE 4:**
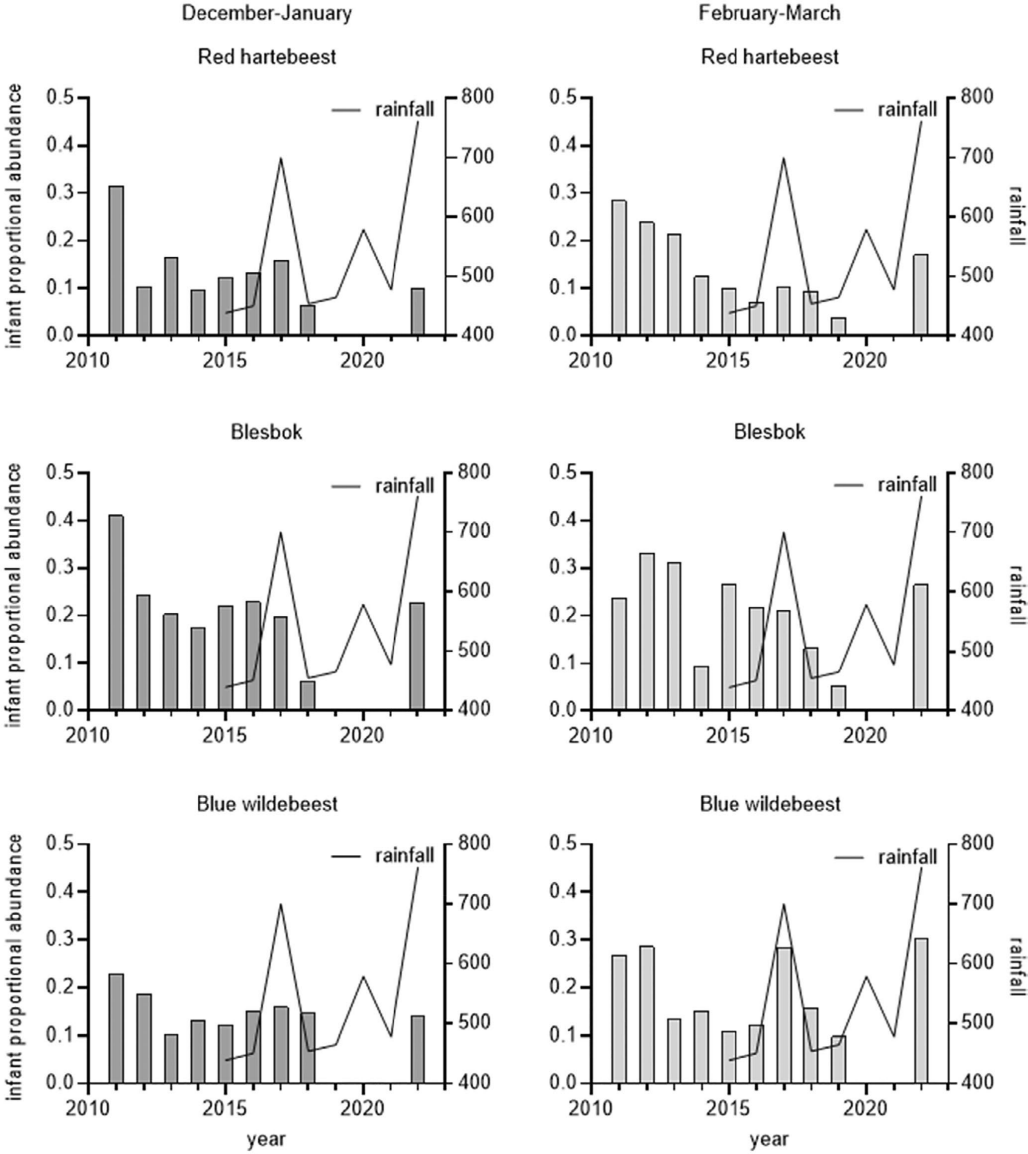
Temporal trends in young‐to‐adult ratios for three study species at Telperion NR and average rainfall during the same study period (2002–2023) for an/Feb and March/April.

Demographic responses were evaluated for the three species with available age structure data (Figure 4). Limitations in data collection during the late dry season prevented a detailed assessment of its effect on herd composition. During the February–March surveys, all species showed some level of demographic response. For red hartebeest, density‐dependent recruitment was also evident in the December–January period. Temporal trends in young‐to‐adult ratios for hartebeest and blesbok showed a distinct decline, particularly during February–March (Figure 4, right column). The trend is less apparent for wildebeest. The 2023 data indicated an increase in young‐to‐adult ratios, likely due to above‐average precipitation, with a high sample size reducing sampling error. Samples sizes for each sampling occasion are summarized in Table S1.

## 4 Discussion

Overcompensatory population growth near equilibrium density is typical for large herbivores (Fowler 1981), particularly in areas with low variation in annual precipitation (Sinclair 2003). A sigmoid population trajectory would be associated with the Ricker or theta models. This study suggests density‐dependent regulation in large herbivore populations at Telperion NR but evidence is lacking for some species. Strong evidence for density dependence was evident only for waterbuck (> 2 delta‐AICc). Wildebeest were the only species showing a linear density‐dependant response but with strong competition from other models (< 2 delta AICc). Considering that blue wildebeest are well adapted to forage on grazing lawns (Owen‐Smith 2002), found lower down the catena in the study area, it is surprising that they did not show a curvilinear density dependence. Evidence for density dependence among zebra was not convincing either, with the curvilinear density dependence closely tied with the geometric model. Red hartebeest displayed quasi‐equilibrium dynamics over most of the time series, suggesting resource limitations at lower overall consumer biomass early on, and perhaps preceding the time when aerial surveys commenced. Their numbers showed a decline towards the end, suggesting possible density‐dependent responses but statistical support is lacking. Blesbok numbers increased four‐fold but did not exhibit a clear sigmoidal trajectory. Their comparatively small body size imparts low per capita forage requirements, which favors them in open grassland habitats. The apparent decline in population growth towards the end of the time series (Figure 2) hints at the presence of density dependence but requires additional data to confirm this.

The study species differentially contributed to consumer biomass, reflecting their varied feeding ecologies. Red hartebeest and waterbuck, with more specialized diets, had lower biomass compared to generalist feeders like zebra and wildebeest. Zebra are hindgut fermenters and consequently more tolerant of low‐quality forage (Owen‐Smith 2002). Their numerical dominance is therefore consistent with our expectations. The relatively low biomass of waterbuck and red hartebeest is consistent with earlier studies (Becker et al. 2021; Owen‐Smith 2021) and our expectations. Waterbuck exhibited increased abundance variability over time, suggesting fluctuating resource availability with rising consumer biomass. Their need for high‐protein and high‐water forage (Becker et al. 2021) makes them susceptible to resource competition among con‐specifics. Similarly, red hartebeest's preference for relatively undisturbed habitat (Murray and Brown 1993; Mariotti et al. 2020) predisposes them to competition from conspecifics (Hibert et al. 2010). While the time series data is viewed from the perspective of intra‐specific competition, competition among species most likely occurs concurrently, with all species not equally affected e.g., low densities of hartebeest and waterbuck most likely stem from competition with con‐specifics. Interspecific competition is hard to demonstrate though (Zini, Wäber, and Dolman 2023). The rising densities of wildebeest and zebra did not evoke a clear numerical response from hartebeest and waterbuck. This, plausibly, might have occurred at a time preceding the study period.

Demographic responses to resource constraints generally follow a predicted sequence among ungulates, with infant survival, rather than reduced birth rate as the first response expected under resource limitation (Sæther 1997; Gaillard et al. 2000). Our findings support this notion. Red hartebeest, blesbok, and wildebeest showed a decline in young‐to‐adult ratios particularly in the February–March period, indicating a stronger influence of resource limitations on survival than reduced birth rates. February–March corresponds to the late wet season. A strong recruitment response during this period suggests severe resource limitations early in the seasonal cycle. The fact that such an early decline in the young‐to‐adult ratio does not lead to a decline in overall numbers (except for Red Hartebeest at the end of the study period) cannot be explained.

The low relative abundance and fluctuating trend of the red hartebeest population could also suggest predation as a possible regulatory mechanism. Predation is an important regulating mechanism among smaller ungulate species (Hopcraft, Olff, and Sinclair 2010). While hartebeest are medium‐sized antelope, they hide their young and could thus be predisposed to high predation risk (Klare et al. 2010). However, evidence from the high young‐to‐adult ratios for hartebeest in earlier years when the overall prey biomass was comparatively low, likely negates this explanation. If any, one would expect top‐down regulation at low overall prey biomass rather than at high consumer biomass densities (Skogland 1991). The limited demographic data indicated that recruitment among the red hartebeest (Feb‐March young: adult ratios) was similar to other species early on in the time series, followed by a distinct low young: adult ratio coinciding with the downward trajectory in abundance. An alternative plausible explanation, however, is that the survival of the young is highly dependent on suitable habitat to conceal their young from predators. The establishment of extensive grazing lawns at Telperion over time possibly means diminishing suitable habitat for the concealment of young. The temporal dynamics of blesbok further illustrate the notion of predation as a potential regulating mechanism. The prolonged period of low growth early on in the time series, followed by a pronounced exponential growth phase suggests an apparent Allee effect (Mooring et al. 2004). Unfortunately, herd composition data for this period is lacking, and our argument remains speculative. Similarly, it is impossible to discount predation's potential impact on adult hartebeest. However, as indicated in the methods section, leopard densities are deemed too low to exert top‐down regulation.

Density‐dependent regulation appears more prevalent in environments with moderate rainfall, like Telperion NR, where annual precipitation averages 654 mm. While density dependence could not be demonstrated across all species, the overall large herbivore biomass stabilized close to predictions by Coe, Cumming, and Phillipson (1976). Bottom‐up regulation therefore appears to be a reasonable inference for the study site. The apparent leveling off somewhat below the predictions by Coe, Cumming, and Phillipson (1976) could result from undercounting during surveys. Other reasons yet to be explored are equally possible. Note that the spatial scale of the current study is small relative to those study sites used by Coe, Cumming, and Phillipson (1976). The reader is referred to Hempson et al. (2015) for a comprehensive treatment of this topic. Despite its small size (~13,000 ha), Telperion NR showed resilience against prolonged grazing, with grazing lawns playing a key role in species persistence (Verweij et al. 2006). Our findings provide insight into dystrophic grassland resilience to prolonged heavy grazing regimes but only time will tell whether the current non‐removal policy is sustainable. The current temporal scale allows capturing a density‐dependent response but might be insufficient for capturing long‐term consumer‐resource dynamics (Illius and O'Connor 1999; Vetter 2005). Our interpretations of the observed temporal trends and long‐term sustainability therefore require caution.

In summary, our expectations were only partially met: (1) evidence of density dependence could not be found across all species; (2) the theta logistic model, was not best supported by the zebra population data, but competed well with the geometric model; (3) the consumer biomass qualitatively hints at leveling off close to an equilibrium density predicted by earlier authors (Coe, Cumming, and Phillipson 1976); (4) zebra dominate the ungulate assemblage numerically; (5) qualitatively calf recruitment appeared to decline with increasing consumer biomass.

## Author Contributions

**Cornelius J. Louw:** conceptualization (lead), data curation (lead), investigation (equal), validation (equal), writing – original draft (lead), writing – review and editing (equal). **Jason P. Marshal:** conceptualization (equal), formal analysis (lead), investigation (equal), methodology (lead), software (lead), validation (equal), writing – review and editing (equal). **Francesca Parrini:** conceptualization (equal), data curation (equal), investigation (equal), methodology (equal), validation (equal), visualization (equal), writing – review and editing (equal).

## Conflicts of Interest

The authors declare no conflicts of interest.

## Supporting information


Data S1.

Data S2.

Data S3.

Data S4.

## Data Availability

All data and R scripts used for analyses are provided as supplemental material.
