# Supplementary material for: Density Dependence in Large Herbivores Inhabiting an Insular Nature Reserve
Source: Ecol Evol. 2024 Dec 4;14(12):e70689. doi: 10.1002/ece3.70689 (PMC11617638; doi:10.1002/ece3.70689)
Supplement: Supplementary file 1 — Data S1. [file ECE3-14-e70689-s003.docx]

Supplementary material for:

Louw CJ, Marshal JP, Parrini F. Density dependence in large herbivores inhabiting an insular nature reserve.

Workspace set-up

**library**(IPMbook)

**library**(jagsUI)

**library**(tidyverse)

**library**(gridExtra)

**library**(AICcmodavg)

**library**(MCMCvis)

Geometric Bayesian state-space model

cat(file="model-geo.txt", "

model {

## priors ##

# ecological model

sig.llam ~ dunif(0, 1) # Prior for sd of growth rate

sig2.llam <- pow(sig.llam, 2)

tau.llam <- pow(sig.llam, -2)

rmax ~ dnorm(0,0.0001)I(-5,5)

# observation process

sig.y ~ dunif(0.1, 1000) # Prior for sd of observation process

sig2.y <- pow(sig.y, 2)

tau.y <- pow(sig.y, -2)

# model for the initial population size

N[1] ~ dunif(0, 500)

## likelihood ##

# ecological model: abundance over time

for (t in 1:(T-1)){

llam[t] ~ dnorm(r[t], tau.llam)

r[t] <- rmax

N[t+1] <- N[t] * exp(llam[t])

}

# observation process

for (t in 1:T){

y[t] ~ dnorm(N[t], tau.y)

}

}

")

Set up data for JAGS processing

telperionData <- read_csv(file = 'TelperionWorking.csv') *# data set containing raw counts*

y.bles <- telperionData$bles

y.bluw <- telperionData$bluw

y.hart <- telperionData$hart

y.watb <- telperionData$watb

y.zeb <- telperionData$zeb

*# bundle data for JAGS*

data.bles <- list(y = y.bles, T = length(y.bles))

data.bluw <- list(y = y.bluw, T = length(y.bluw))

data.hart <- list(y = y.hart, T = length(y.hart))

data.watb <- list(y = y.watb, T = length(y.watb))

data.zeb <- list(y = y.zeb, T = length(y.zeb))

*# initial values*

inits <- **function**(){list(sig.llam=runif(1, 0, 1))}

*# parameters monitored*

params <- c('lam', 'llam', 'sig2.y', 'sig2.llam', 'sig.y', 'sig.llam', 'N',

'rmax', 'mean.lam')

*# MCMC settings*

ni <- 1000000; nb <- 500000; nc <- 3; nt <- 100; na <- 5000

Run the state-space model in JAGS

Output includes corrected counts and rr (= llam) for each species.

*# blesbok*

out.bles.geo <- jags(data.bles, inits, params, "model-geo.txt", n.iter=ni,

n.burnin=nb, n.chains=nc, n.thin=nt, n.adapt=na,

parallel=TRUE)

save(out.bles.geo, file = 'out-bles-geo.RData')

*# blue wildebeest*

out.bluw.geo <- jags(data.bluw, inits, params, "model-geo.txt", n.iter=ni,

n.burnin=nb, n.chains=nc, n.thin=nt, n.adapt=na,

parallel=TRUE)

save(out.bluw.geo, file = 'out-bluw-geo.RData')

*# hartebeest*

out.hart.geo <- jags(data.hart, inits, params, "model-geo.txt", n.iter=ni,

n.burnin=nb, n.chains=nc, n.thin=nt, n.adapt=na,

parallel=TRUE)

save(out.hart.geo, file = 'out-hart-geo.RData')

*# waterbuck*

out.watb.geo <- jags(data.watb, inits, params, "model-geo.txt", n.iter=ni,

n.burnin=nb, n.chains=nc, n.thin=nt, n.adapt=na,

parallel=TRUE)

save(out.watb.geo, file = 'out-watb-geo.RData')

*# plains zebra*

out.zeb.geo <- jags(data.zeb, inits, params, "model-geo.txt", n.iter=ni,

n.burnin=nb, n.chains=nc, n.thin=nt, n.adapt=na,

parallel=TRUE)

save(out.zeb.geo, file = 'out-zeb-geo.RData')

Check MCMCs and posterior distributions

*# to load saved output files, if we don't want to rerun MCMCs every time*

load('out-bles-geo.RData')

load('out-bluw-geo.RData')

load('out-hart-geo.RData')

load('out-watb-geo.RData')

load('out-zeb-geo.RData')

*# blesbok*

*#traceplot(out.bles.geo)*

*#densityplot(out.bles.geo)*

*#print(out.bles.geo, 3)*

*#MCMCsummary(out.bles.geo, params = c('rmax', 'mean.lam'), round = 4)*

*# blue wildebeest*

*#traceplot(out.bluw.geo)*

*#densityplot(out.bluw.geo)*

*#print(out.bluw.geo, 3)*

*#MCMCsummary(out.bluw.geo, params = c('rmax', 'mean.lam'), round = 4)*

*# hartebeest*

*#traceplot(out.hart.geo)*

*#densityplot(out.hart.geo)*

*#print(out.hart.geo, 3)*

*#MCMCsummary(out.hart.geo, params = c('rmax', 'mean.lam'), round = 4)*

*# waterbuck*

*#traceplot(out.watb.geo)*

*#densityplot(out.watb.geo)*

*#print(out.watb.geo, 3)*

*#MCMCsummary(out.watb.geo, params = c('rmax', 'mean.lam'), round = 4)*

*# plains zebra*

*#traceplot(out.zeb.geo)*

*#densityplot(out.zeb.geo)*

*#print(out.zeb.geo, 3)*

*#MCMCsummary(out.zeb.geo, params = c('rmax', 'mean.lam'), round = 4)*

Compare geometric, Ricker and theta-logistic models

Model parameter estimates were calculated iteratively from the Markov chain values for llam and corrected counts.

mod.names <- c('Geometric', 'Ricker', 'Theta')

*## blesbok ##*

rr <- out.bles.geo$mean$llam

NN <- out.bles.geo$mean$N[1:21]

bles.mod <- list()

bles.mod[[1]] <- nls(rr ~ rmax, start = list(rmax = 0.03))

bles.mod[[2]] <- nls(rr ~ rmax + b*NN, start = list(rmax = 0.25, b = -0.016))

bles.mod[[3]] <- nls(rr ~ rmax * (1 - I(NN/K)^theta),

start = list(rmax = 0.105, K = 560, theta = 6))

aictab(cand.set = bles.mod, modnames = mod.names)

##

## Model selection based on AICc:

##

## K AICc Delta_AICc AICcWt Cum.Wt LL

## Geometric 2 -35.51 0.00 0.98 0.98 16.76

## Ricker 3 -26.77 8.74 0.01 1.00 17.09

## Theta 4 -24.35 11.16 0.00 1.00 17.43

*# geometric lowest AICc*

N.sims <- out.bles.geo$sims.list$N

ll.sims <- out.bles.geo$sims.list$llam

n.iter <- dim(N.sims)[1]

nlc <- nls.control(maxiter = 1000)

coeffs <- rep(NA, times = n.iter)

**for** (i **in** 1:n.iter){

rr <- ll.sims[i,]

NN <- N.sims[i,1:21]

model1 <- nls(rr ~ rmax, start = list(rmax = 0.03))

coeffs[i] <- coefficients(model1)

}

*# rmax*

median(coeffs)

## [1] 0.07373087

quantile(coeffs, probs = c(0.025, 0.975), na.rm = T)

## 2.5% 97.5%

## 0.06087108 0.09941464

*## blue wildebeest ##*

rr <- out.bluw.geo$mean$llam

NN <- out.bluw.geo$mean$N[1:21]

bluw.mod <- list()

bluw.mod[[1]] <- nls(rr ~ rmax, start = list(rmax = 0.03))

bluw.mod[[2]] <- nls(rr ~ rmax + b*NN, start = list(rmax = 0.25, b = -0.016))

bluw.mod[[3]] <- nls(rr ~ rmax * (1 - I(NN/K)^theta),

start = list(rmax = 0.1, K = 800, theta = 3))

aictab(cand.set = bluw.mod, modnames = mod.names)

##

## Model selection based on AICc:

##

## K AICc Delta_AICc AICcWt Cum.Wt LL

## Ricker 3 -79.27 0.00 0.52 0.52 43.34

## Theta 4 -78.07 1.21 0.29 0.81 44.28

## Geometric 2 -77.26 2.02 0.19 1.00 37.63

*# Ricker lowest AICc*

N.sims <- out.bluw.geo$sims.list$N

ll.sims <- out.bluw.geo$sims.list$llam

n.iter <- dim(N.sims)[1]

nlc <- nls.control(maxiter = 1000)

coeffs <- matrix(NA, nrow = n.iter, ncol = 2)

**for** (i **in** 1:n.iter){

rr <- ll.sims[i,]

NN <- N.sims[i,1:21]

model1 <- nls(rr ~ rmax + b*NN, start = list(rmax = 0.25, b = -0.016))

coeffs[i,] <- coefficients(model1)

}

*# rmax*

median(coeffs[,1])

## [1] 0.149171

quantile(coeffs[,1], probs = c(0.025, 0.975), na.rm = T)

## 2.5% 97.5%

## 0.05701115 0.24998159

*# b*

median(coeffs[,2])

## [1] -0.0001713003

quantile(coeffs[,2], probs = c(0.025, 0.975), na.rm = T)

## 2.5% 97.5%

## -3.357729e-04 -1.643679e-05

*# theta a close second*

coeffs <- matrix(NA, nrow = n.iter, ncol = 3)

**for** (i **in** 1:n.iter){

**tryCatch**({

rr <- ll.sims[i,]

NN <- N.sims[i,1:21]

model1 <- nls(rr ~ rmax * (1 - I(NN/K)^theta), control = nlc,

start = list(rmax = 0.1, K = 800, theta = 3))

coeffs[i,] <- coefficients(model1)},

error=**function**(e){})

}

*# rmax*

median(coeffs[,1], na.rm = T)

## [1] 0.09296188

quantile(coeffs[,1], probs = c(0.025, 0.975), na.rm = T)

## 2.5% 97.5%

## 0.05159907 0.26678197

*# K*

median(coeffs[,2], na.rm = T)

## [1] 800.708

quantile(coeffs[,2], probs = c(0.025, 0.975), na.rm = T)

## 2.5% 97.5%

## 730.0914 1224.6627

*# theta*

median(coeffs[,3], na.rm = T)

## [1] 4.027647

quantile(coeffs[,3], probs = c(0.025, 0.975), na.rm = T)

## 2.5% 97.5%

## 0.4911188 35.3520324

*## hartebeest ##*

rr <- out.hart.geo$mean$llam

NN <- out.hart.geo$mean$N[1:21]

hart.mod <- list()

hart.mod[[1]] <- nls(rr ~ rmax, start = list(rmax = 0.03))

hart.mod[[2]] <- nls(rr ~ rmax + b*NN, start = list(rmax = 0.25, b = -0.016))

*#hart.mod[[3]] <- nls(rr ~ rmax * (1 - I(NN/K)^theta),*

*# start = list(rmax = 0.0, K = 180, theta = 1))*

*## theta model doesn't work:*

*## singular gradient matrix at initial parameter estimates*

aictab(cand.set = hart.mod, modnames = c('Geometric', 'Ricker'))

##

## Model selection based on AICc:

##

## K AICc Delta_AICc AICcWt Cum.Wt LL

## Geometric 2 -47.74 0.00 0.99 0.99 22.87

## Ricker 3 -38.81 8.93 0.01 1.00 23.11

*# geometric lower AICc*

N.sims <- out.hart.geo$sims.list$N

ll.sims <- out.hart.geo$sims.list$llam

n.iter <- dim(N.sims)[1]

nlc <- nls.control(maxiter = 1000)

coeffs <- rep(NA, times = n.iter)

**for** (i **in** 1:n.iter){

rr <- ll.sims[i,]

NN <- N.sims[i,1:21]

model1 <- nls(rr ~ rmax, start = list(rmax = 0.03))

coeffs[i] <- coefficients(model1)

}

*# rmax*

median(coeffs)

## [1] -0.02666996

quantile(coeffs, probs = c(0.025, 0.975), na.rm = T)

## 2.5% 97.5%

## -0.062430047 0.003326507

*## plains zebra ##*

rr <- out.zeb.geo$mean$llam

NN <- out.zeb.geo$mean$N[1:21]

zeb.mod <- list()

zeb.mod[[1]] <- nls(rr ~ rmax, start = list(rmax = 0.03))

zeb.mod[[2]] <- nls(rr ~ rmax + b*NN, start = list(rmax = 0.25, b = -0.016))

zeb.mod[[3]] <- nls(rr ~ rmax * (1 - I(NN/K)^theta),

start = list(rmax = 0.17, K = 1000, theta = 3))

aictab(cand.set = zeb.mod, modnames = mod.names)

##

## Model selection based on AICc:

##

## K AICc Delta_AICc AICcWt Cum.Wt LL

## Geometric 2 -41.93 0.00 0.45 0.45 19.96

## Theta 4 -41.91 0.02 0.45 0.90 26.20

## Ricker 3 -38.89 3.04 0.10 1.00 23.15

*# geometric lowest AICc*

N.sims <- out.zeb.geo$sims.list$N

ll.sims <- out.zeb.geo$sims.list$llam

n.iter <- dim(N.sims)[1]

nlc <- nls.control(maxiter = 1000)

coeffs <- rep(NA, times = n.iter)

**for** (i **in** 1:n.iter){

rr <- ll.sims[i,]

NN <- N.sims[i,1:21]

model1 <- nls(rr ~ rmax, start = list(rmax = 0.03))

coeffs[i] <- coefficients(model1)

}

*# rmax*

median(coeffs)

## [1] 0.04709023

quantile(coeffs, probs = c(0.025, 0.975), na.rm = T)

## 2.5% 97.5%

## 0.03124395 0.06696720

*# theta a close second*

coeffs <- matrix(NA, nrow = n.iter, ncol = 3)

**for** (i **in** 1:n.iter){

**tryCatch**({

rr <- ll.sims[i,]

NN <- N.sims[i,1:21]

model1 <- nls(rr ~ rmax * (1 - I(NN/K)^theta), control = nlc,

start = list(rmax = 0.17, K = 1000, theta = 3))

coeffs[i,] <- coefficients(model1)},

error=**function**(e){})

}

*# rmax*

median(coeffs[,1], na.rm = T)

## [1] 0.1106881

quantile(coeffs[,1], probs = c(0.025, 0.975), na.rm = T)

## 2.5% 97.5%

## 0.06536959 0.21499295

*# K*

median(coeffs[,2], na.rm = T)

## [1] 1054.684

quantile(coeffs[,2], probs = c(0.025, 0.975), na.rm = T)

## 2.5% 97.5%

## 968.1344 1154.0918

*# theta*

median(coeffs[,3], na.rm = T)

## [1] 5.227367

quantile(coeffs[,3], probs = c(0.025, 0.975), na.rm = T)

## 2.5% 97.5%

## 1.748786 15.552239

*## waterbuck ##*

rr <- out.watb.geo$mean$llam

NN <- out.watb.geo$mean$N[1:21]

watb.mod <- list()

watb.mod[[1]] <- nls(rr ~ rmax, start = list(rmax = 0.03))

watb.mod[[2]] <- nls(rr ~ rmax + b*NN, start = list(rmax = 0.25, b = -0.016))

watb.mod[[3]] <- nls(rr ~ rmax * (1 - I(NN/K)^theta),

start = list(rmax = 0.1, K = 80, theta = 1))

aictab(cand.set = watb.mod, modnames = mod.names)

##

## Model selection based on AICc:

##

## K AICc Delta_AICc AICcWt Cum.Wt LL

## Theta 4 -56.71 0.00 0.97 0.97 33.60

## Geometric 2 -49.67 7.04 0.03 1.00 23.83

## Ricker 3 -42.41 14.30 0.00 1.00 24.91

*# theta lowest AICc*

N.sims <- out.watb.geo$sims.list$N

ll.sims <- out.watb.geo$sims.list$llam

n.iter <- dim(N.sims)[1]

nlc <- nls.control(maxiter = 1000)

coeffs <- matrix(NA, nrow = n.iter, ncol = 3)

**for** (i **in** 1:n.iter){

**tryCatch**({

rr <- ll.sims[i,]

NN <- N.sims[i,1:21]

model1 <- nls(rr ~ rmax * (1 - I(NN/K)^theta), control = nlc,

start = list(rmax = 0.04, K = 56, theta = 20))

coeffs[i,] <- coefficients(model1)},

error=**function**(e){})

}

*# rmax*

median(coeffs[,1], na.rm = T)

## [1] 0.05673382

quantile(coeffs[,1], probs = c(0.025, 0.975), na.rm = T)

## 2.5% 97.5%

## 0.01383667 1.31635706

*# K*

median(coeffs[,2], na.rm = T)

## [1] 81.39796

quantile(coeffs[,2], probs = c(0.025, 0.975), na.rm = T)

## 2.5% 97.5%

## 60.5709 117.1809

*# theta*

median(coeffs[,3], na.rm = T)

## [1] 8.969569

quantile(coeffs[,3], probs = c(0.025, 0.975), na.rm = T)

## 2.5% 97.5%

## 0.3527357 115.5982659
