# Supplementary material for: Density Dependence in Large Herbivores Inhabiting an Insular Nature Reserve
Source: Ecol Evol. 2024 Dec 4;14(12):e70689. doi: 10.1002/ece3.70689 (PMC11617638; doi:10.1002/ece3.70689)
Supplement: Supplementary file 2 — Data S2. [file ECE3-14-e70689-s004.docx]

**Appendix**

**Table S1**: Sample sizes of herd composition data for red hartebeest, blesbok, and blue wildebeest at Telperion NR collected from 2011 to 2022.

| Species | Year | Sample size | |
| --- | --- | --- | --- |
| Red hartebeest |  | Dec/Jan | Feb/March |
|  | 2011 | 206 | 163 |
|  | 2012 | 150 | 291 |
|  | 2013 | 71 | 176 |
|  | 2014 | 56 | 72 |
|  | 2015 | 55 | 22 |
|  | 2016 | 17 | 60 |
|  | 2017 | 73 | 43 |
|  | 2018 |  | 58 |
|  | 2019 |  | 83 |
|  | 2020 |  |  |
|  | 2021 |  |  |
|  | 2022 | 33 | 144 |
| Blesbok | 2011 | 550 | 335 |
|  | 2012 | 515 | 482 |
|  | 2013 | 89 | 560 |
|  | 2014 | 94 | 104 |
|  | 2015 | 249 | 43 |
|  | 2016 | 129 | 235 |
|  | 2017 | 225 | 189 |
|  | 2018 | 88 | 198 |
|  | 2019 |  | 245 |
|  | 2020 |  |  |
|  | 2021 |  |  |
|  | 2022 | 571 | 492 |
| Blue wildebeest | 2011 | 721 | 633 |
|  | 2012 | 504 | 523 |
|  | 2013 | 529 | 244 |
|  | 2014 | 413 | 367 |
|  | 2015 | 680 | 396 |
|  | 2016 | 609 | 638 |
|  | 2017 | 476 | 616 |
|  | 2018 | 63 | 493 |
|  | 2019 |  | 159 |
|  | 2020 |  |  |
|  | 2021 |  |  |
|  | 2022 | 854 | 731 |
